# Trehalose Contributes to Gamma-Linolenic Acid Accumulation in *Cunninghamella echinulata* Based on *de Novo* Transcriptomic and Lipidomic Analyses

**DOI:** 10.3389/fmicb.2018.01296

**Published:** 2018-06-15

**Authors:** Shue Li, Qiang Yue, Shuai Zhou, Jing Yan, Xiaoyu Zhang, Fuying Ma

**Affiliations:** ^1^Key Laboratory of Molecular Biophysics of MOE, College of Life Science and Technology, Huazhong University of Science and Technology, Wuhan, China; ^2^Hubei Key Laboratory of Lipid Chemistry and Nutrition, Wuhan, China

**Keywords:** filamentous fungi, gamma-linolenic acid, trehalose, transcriptome, phospholipidome, phosphatidylinositol

## Abstract

Gamma-linolenic acid (GLA) is essential for the well-being of humans and other animals. People may lack GLA because of aging or diseases, and thus, dietary supplements or medical reagents containing GLA-enriched lipids are in demand. *Cunninghamella echinulata* is a potential GLA-producing strain. Interestingly, we found that the GLA content of *C. echinulata* FR3 was up to 21% (proportion of total lipids) when trehalose was used as a carbon source, significantly higher than the 13% found when glucose was used. Trehalose is quite common and can be accumulated in microorganisms under stress conditions. However, little information is available regarding the role of trehalose in GLA synthesis and accumulation. Our study aimed to understand how the metabolism of *C. echinulata* responds to trehalose as a carbon source for GLA and lipid biosynthesis. We profiled the major sugars, fatty acids, phospholipids, and gene transcripts of *C. echinulata* FR3 grown in trehalose medium with glucose as a control by *de novo* transcriptomics, lipidomics, and other methods. The results showed that trehalose could influence the expression of desaturases and that the GLA proportion increased because of delta-6 desaturase upregulation. The increased GLA was transferred to the extracellular environment through the active PI ion channel, which prefers polyunsaturated acyl chains. At the same time, trehalose might prevent GLA from peroxidation by forming a trehalose-polyunsaturated fatty acid (PUFA) complex. Our study provides new insights into the functions of trehalose in GLA accumulation.

## Introduction

Gamma-linolenic acid (*all-cis*-6,9,12-octadecatrienoic acid, C18:3, GLA), an essential n-6 polyunsaturated fatty acid (PUFA), is a minor component of animal and human fats and an intermediate in the biosynthesis of biologically active hormones (prostaglandins, thromboxanes, and leukotrienes) (Horrobin, 1992). GLA can increase epidermal levels of eicosanoid precursors and prevent or alleviate hardening of the arteries and heart disease (Guil-Guerrero et al., 2001; Sergeant et al., 2016). Many health and dietary studies claim that GLA prevents or alleviates a variety of human and animal diseases. GLA-containing oil is effective for curing rheumatoid arthritis (Jónasdóttir et al., 2017) and ameliorating neuroinflammation (Youn et al., 2018), and high blood pressure (Kapoor and Nair, 2005).

Gamma-linolenic acid can be synthesized from linoleic acid (LA) via delta-6 desaturase desaturation, but neither LA nor GLA can be synthesized *de novo* from dietary oleic acid (Ratledge, 2016). If desaturation by delta-6 desaturase is impaired for any reason, particularly in aging people or those with lipid metabolism disorders, the supply of the resulting metabolites may be inadequate for normal function (Horrobin, 1993). Commercial quantities of GLA are produced from the seed oils of borage (*Borago officinalis*) (20–25% GLA), blackcurrant (*Ribes nigrum*) (15–17% GLA), and evening primrose (*Oenothera biennis*) (9–12% GLA) (Guil-Guerrero et al., 2001). Evening primrose oil remains the primary source, but the production of evening primrose oil is restricted by season, water availability and the scale of farming (Heuer et al., 2002). Microbial lipids have been considered as sources of abundant PUFAs for the food and medical industries (Bellou et al., 2016). Among microbes, one fungus with GLA-enriched lipids, *Cunninghamella echinulata*, has attracted additional attention in the biotechnology industry. Many attempts have been dedicated to optimizing its GLA content or total lipid content (Fakas et al., 2008; Al-Hawash et al., 2017). According to these studies, glucose is considered the best carbon source for lipid synthesis (Chatzifragkou et al., 2010). Furthermore, based on our prior studies, 5% glucose is suitable for unsaturated fatty acid production by *C. echinulata* FR3 (Al-Hawash et al., 2017). Surprisingly, we found that the GLA proportion in *C. echinulata* FR3 was significantly higher when using trehalose as a carbon source than when using 5% glucose.

Trehalose is quite common in fungi, bacteria, plants and invertebrates, but it is not found in vertebrates (Elbein, 1974; Argüelles, 2014). This non-reducing disaccharide contains an α,α-1,1-glucoside bond between two α-glucose units and is extremely stable (Tereshina, 2005; Collins et al., 2018). Some researchers have reported that fungi could endogenously produce trehalose as a stabilizer and protectant of membranes or proteins in response to stress conditions, such as heat, cold, acidification, salinity, ROS stress and cell wall damage (Liu et al., 2016; Shleeva et al., 2017; Zeidler et al., 2017). Accordingly, the role and application of exogenous trehalose have mainly been discussed in plants (Ma et al., 2013; Govind et al., 2016). However, the role of exogenous trehalose in fungi is associated with carbon and energy reserves, stress protection (Plourde-Owobi et al., 2000), and manner of growth (dimorphism) (Lucio et al., 2000).

Some stress conditions are known to be beneficial to lipid metabolism. To date, there has been a lack of detailed analyses of the correlation between trehalose and PUFA *in vivo*, especially the physiological role of trehalose in GLA synthesis and accumulation in *C. echinulata*. Lipidomics can quantify spatial and temporal alterations in the content and composition of lipid molecular species (Brügger, 2014), and suitable transcriptome data can provide valuable information. Therefore, our study focused on how the metabolism of *C. echinulata* responds to trehalose as a carbon source and how GLA accumulates, and we coupled lipidomics with transcriptomics for further investigation.

## Materials and Methods

### Strain and Culture Conditions

*Cunninghamella echinulata* FR3 was preserved on potato dextrose agar (PDA) slants at 4°C and transferred onto a fresh PDA slant before use. Mycelial plugs cut from the slant were transferred into potato dextrose broth (PDB). After 2 days of incubation at 25°C and 150 rpm, 10 mL of inoculum was transferred into fresh PDB to incubate for 2 days as a new inoculum.

*Cunninghamella echinulata* FR3 was grown in fermentation medium for 8 days. The fermentation medium contained (per L): 200 g potato extract with 20 g trehalose (2% trehalose group), 50 g trehalose (5% trehalose group), or 50 g glucose (5% glucose group). After incubation, the mycelia were collected for lipidomic and transcriptomic analyses. All cultivation was conducted at 25°C and 150 rpm with an initial pH of 6.0. The overall experimental strategy is shown in Supplementary Figure S1.

### Sugar Test

To determine sugar and sugar alcohol contents, the washed mycelium was dried at a constant temperature, and the fermentation broth was freeze-dried. Samples (100 mg or 1 mL) were extracted in 10 ml of ultra-pure water at 100°C for 2 h, passed through a 0.45-μm filter (Millipore, Bedford, MA, United States), and then analyzed using an IC2500 HPAEC-PAD system with a GP50 quaternary gradient pump, an LC30 column oven, an EG50 automatic eluent generator, an ED50 electrochemical detector and a Dionex CarboPac MA1 column (Dionex, Sunnyvale, CA, United States). The column temperature was 30°C, and the mobile phase was 480 mM NaOH solution at a flow rate of 0.4 mL/min. Six standard external substances were used, including arabitol, mannitol, trehalose, erythritol, galactose, and glucose (Sigma, United States). Each standard substance was dissolved in deionized water and diluted in a series of standard solutions to obtain a calibration curve (Zhou et al., 2012).

### Dry Weight Estimation and Total Lipid Extraction and Analysis

Fungal mycelia were harvested by filtration through Whatman no. 1 filter paper and washed three times with distilled water. The filtered mycelia were dried under vacuum evaporation to a constant weight. The dry biomass was ground to a soft powder using a grinder. Total lipid was extracted from 20.0 mg dry mycelial powder according to the method of Tan (Tan et al., 2017) with minor modification, using 5% (v/v) H_2_SO_4_ instead of 5% (v/v) KOH. Fatty acid methyl esters (FAME) were analyzed by gas chromatography (GC, Agilent 7890A, United States) with a capillary column (FFAP, 30 m, 0.25 mm i.d., 0.25 μm film thickness) (Wei et al., 2008).

### Phospholipid Analysis

Trehalose is known for its involvement in membrane stabilization processes in many living organisms (Elbein et al., 2003), including plants and fungi. Phospholipids (PLs) are quantitatively the most important lipids in all cell and organelle membranes, as biological membranes have a highly dynamic two-dimensional structure, and these lipids form the structural basis of each membrane by forming a bilayer (Takatori et al., 2014). To understand the influence of trehalose and glucose on PLs, we conducted a phospholipidomic analysis.

Prior to sample extraction, 1 μg of PC (24:1/24:1), PI (40:6), PE (12:0/12:0), PG (14:0/14:0), PS (14:0/14:0), and PA (14:0/14:0) from Avanti Polar Lipids (Alabaster, United States), were added as internal standards (ISs) for quantification. Phospholipid extraction was performed using the Bligh and Dyer method with some modifications (Bligh and Dyer, 1959; Wang et al., 2015). After the initial extraction, the dried samples were redissolved in methanol/isopropanol (1:1) solution and then concentrated and filtered through a 0.22 μm pore size polyvinylidene difluoride membrane filter (Millipore Bedford, MA, United States). The cleaned extracts were stored at -80°C before analysis.

Phospholipid analysis was conducted on an Acquity UPLC system (Waters, Ireland) coupled to an AB Sciex 6500 QTRAP triple quadrupole, linear ion trap mass spectrometer. An Acquity UPLC BEH C18, 2.1 × 100 mm; 1.7 μm column (Waters, Ireland) was used for elution. The eluents A (water:methanol:acetonitrile = 21:20:60, v/v/v; 5 mmol/L NH_4_Ac) and B (isopropanol, 5 mmol/L NH_4_Ac) were employed in the electrospray ionization-positive (ESI+) mode. The linear gradient increased from 0 to 20% B over 0.5 min, from 20 to 40% for the next 1 min, from 40 to 60% B during the next 1.5 min, and from 60 to 98% B for the next 10 min, and then immediately ramped to 20% B and maintained for 4 min. Then, 10 μL aliquots of each sample were injected onto the column. The column temperature was kept at 40°C. All samples were kept at 4°C throughout the analysis.

Mass spectrometry (MS) was performed on an AB Sciex 6500 QTRAP triple quadrupole, linear ion trap mass spectrometer equipped with a Turbo V ion source. Lipid mediators were detected in positive electrospray ion (ESI) mode. Curtain gas (CUR), nebulizer gas (GS1), and turbo gas (GS2) were set at 10, 30, and 30 psi, respectively. The electrospray voltage was -4.5 kV, and the turbo ion spray source temperature was 550°C. Phospholipids were analyzed using enhanced MS (EMS) and scheduled multiple reaction monitoring (MRM). Mass spectrometer parameters including the declustering potentials and collision energies were optimized for each analysis. Nitrogen was employed as the collision gas. Analyst 1.6.2 software (Applied Biosystems) was used to acquire and analyze data.

### RNA Extraction, Library Construction, and *de Novo* RNA-Seq

To identify the genes involved in the lipid biosynthesis of *C. echinulata* FR3, RNA libraries were constructed using an Illumina HiSeq 4000 platform in the paired-end 150 bp mode by Novogene (Beijing, China). Total RNA was extracted using TRIzol reagent (Invitrogen, United States). The purity of total RNA was measured in a NanoPhotometer spectrophotometer (Implen, United States). RNA integrity was assessed using the RNA Nano 6000 Assay Kit on the Agilent Bioanalyzer 2100 system (Agilent, United States). A total of 1.5 μg RNA was used as input material for the RNA sample preparations. In this study, six sequencing libraries (5% glucose and 2% trehalose groups, each with three replicates) were generated using the NEBNext Ultra RNA Library Prep Kit for Illumina (NEB, United States) following the manufacturer’s recommendations.

High-quality reads (clean reads) were obtained by removing reads containing adaptor, reads containing poly-N and low-quality reads from the raw data. At the same time, the Q20, Q30, GC content and sequence duplication level of the clean data were calculated. All downstream analysis was based on clean data with high quality. Based on 12 samples (other treatments were not shown in this paper), the total of clean reads from those libraries were pooled and *de novo* assembled into a reference transcriptome using Trinity with 2 for the min_kmer_cov setting and the default settings for all other parameters (Grabherr et al., 2011; Haas et al., 2013).

### Bioinformatics Analysis

The assembled unigenes were annotated by BLASTx(*E*-values ≤ 1.0e-5) (Altschul et al., 1997). Gene functions were based on the following databases: NCBI non-redundant protein sequences (Nr), NCBI non-redundant nucleotide sequences (Nt), Protein family (Pfam), Clusters of Orthologous Groups of proteins (KOG/COG), a manually annotated and reviewed protein sequence database (Swiss-Prot), KEGG Ortholog database (KO), and Gene Ontology (GO) (Kanehisa et al., 2008).

The unigene expression levels were normalized by calculating the reads per kilobase of exon model per million mapped reads (RPKM) (Mortazavi et al., 2008). False discovery rate (FDR) is a method to determine an appropriate P-value threshold when conducting multiple tests. RPKM was used to measure the gene expression levels. For genes with more than one alternative transcript, RNA-seq by Expectation Maximization (RSEM), which is a software package, could choose the longest transcript to calculate the expression level, eliminating the influences of gene length and sequencing differences (Li and Dewey, 2011).

To identify the differentially expressed gene between the 2% trehalose and 5% glucose groups, an analysis was performed using the DESeq R package (1.10.1) (Faherty et al., 2015). The resulting *P*-values were adjusted using Benjamini and Hochberg’s approach for controlling the FDR. Genes with an adjusted *P*-value < 0.05 by DESeq were assigned as differentially expressed.

To predict the biological functions of these differentially expressed genes (DEGs), all DEGs were further subjected to GO enrichment analysis and KEGG pathway enrichment analysis to verify their biological significance. The GO enrichment analysis of the DEGs was implemented by the GOseq R package based on the Wallenius non-central hypergeometric distribution (Young et al., 2010), which can adjust for gene length bias in DEGs. KOBAS software (Mao et al., 2005) was used to test the statistical enrichment of the DEGs in KEGG pathways.

### Confirmation by qRT-PCR

Reverse transcription was performed using the PrimeScript^TM^ RT Reagent Kit with gDNA Eraser (Takara, China). The primers were designed using Oligo 7 (Supplementary Table S1). qRT-PCR was carried out using the SYBR Premix Ex TaqTM Kit (Takara, China) on a StepOnePlus Real-Time PCR System (Applied Biosystems, Carlsbad, CA, United States). PCR conditions were 95°C for 10 min, followed by 40 cycles of 15 s at 95°C and 30 s at 60°C, in a volume of 20 μl. The relative expression levels were calculated using the 2^-ΔΔCt^ method by normalizing the copy numbers of the target genes to those of the internal reference gene (Livak and Schmittgen, 2001).

### Statistical Analysis

Samples were collected in triplicate, and each sample was tested third times. Values are presented as the means ± SD values in the figures. Differences between sample means were analyzed by Fisher’s least significant difference (LSD) test at *p* = 0.05.

## Results

### Growth Curve of *C. echinulata* FR3 and Sugar Content in Mycelium and Fermentation Broth

**FIGURE 1:**
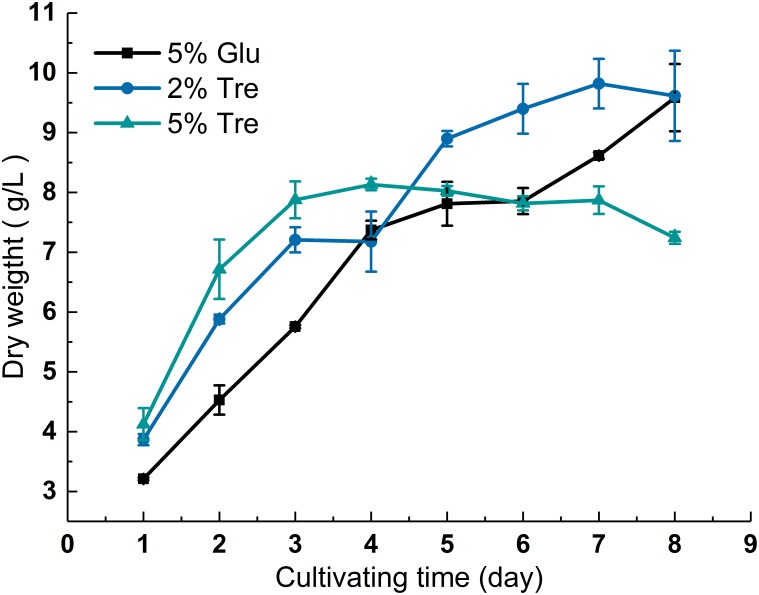
Growth curve of *C. echinulata* FR3 under different sugar concentrations.

The dry biomass of *C. echinulata* FR3 was determined in the presence of 2% trehalose, 5% trehalose, and 5% glucose, and the growth curves are shown in **Figure 1**. The biomass in 2% trehalose (9.39 g/L) was slightly higher than those in 5% trehalose (7.82 g/L) and 5% glucose (8.6 g/L) on the 7th day (**Figure 1**). At the end of the experiment, the dry biomass measurements in 2% trehalose and 5% glucose were higher than that in 5% trehalose. These results verified that trehalose could act as a carbon and energy source for growth (Thompson, 2003), while the higher concentration of trehalose (5% trehalose vs. 2% trehalose) did not yield a higher dry biomass.

**FIGURE 2:**
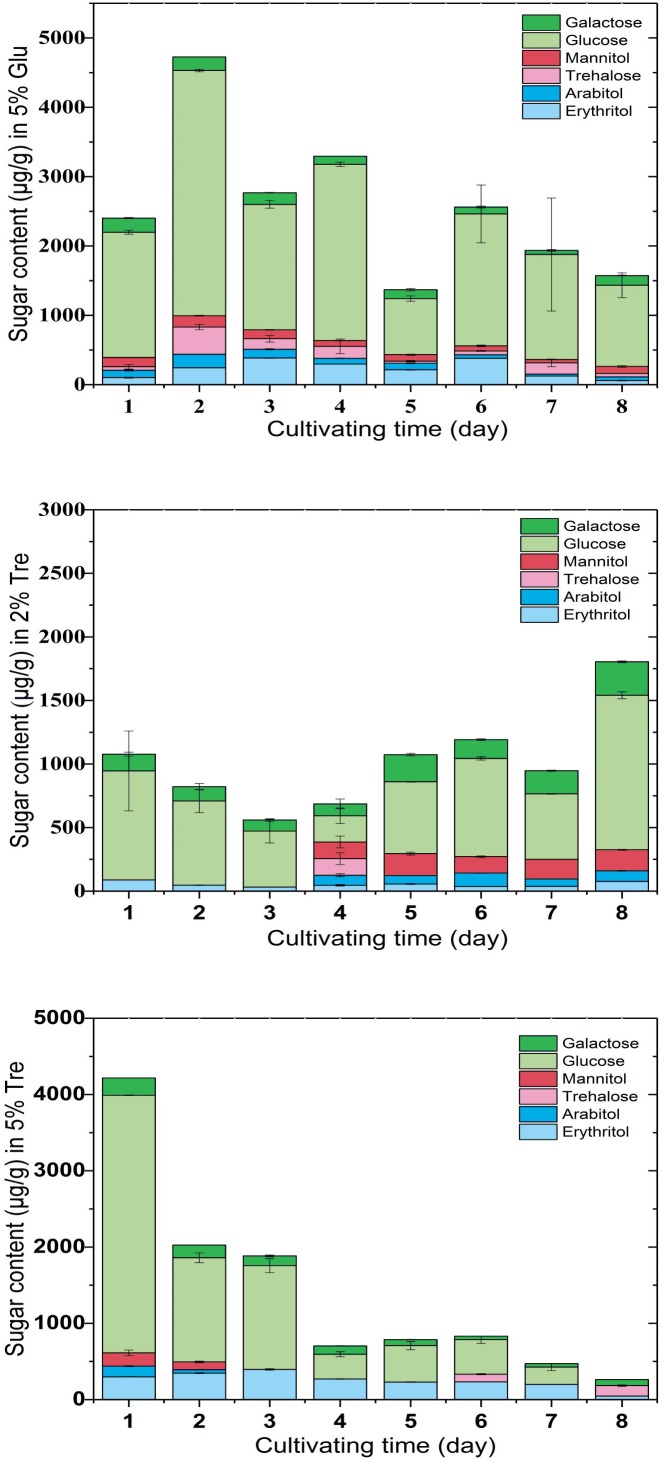
Sugar content in the mycelium of *C. echinulata* FR3 using 5% glucose, 2% trehalose, and 5% trehalose as carbon sources.

To understand the sugar distribution over the time course, we investigated the pattern of sugar contents in the culture supernatant and mycelium. In the culture supernatant, the residual sugar in the 5% glucose medium was 1% on the 8^th^ day (Supplementary Figure S2A), while the residual sugar in the 2% trehalose medium was nearly exhausted (Supplementary Figure S2B). In the mycelium of *C. echinulata* FR3, we found that glucose, erythritol, mannitol, arabitol, trehalose, and galactose were accumulated during growth. These sugars are mainly compatible solutes, which act as protective agents in fungi (Zeidler et al., 2017). In the mycelium grown in 5% glucose medium, a higher concentration of glucose and a lower concentration of trehalose were present, and trehalose was always detected. In the mycelium grown in 2% trehalose medium, the total concentration of sugars was lower than that of the mycelium grown in 5% glucose medium, in which glucose was predominant and trehalose was nearly undetectable, while arabitol, mannitol, and galactose increased from the 4th day to the 8th day. In the mycelium grown in 5% trehalose medium, the total concentration of sugars decreased gradually, and trehalose could be detected in only the later period (**Figure 2**).

The conditions that trigger trehalose accumulation seem to vary, including high sugar (Erasmus et al., 2003), glucose depletion in the medium (Fu et al., 1995), oxidative stress and cell wall damage. When *C. echinulata* FR3 was grown in glucose medium, trehalose was always present in the mycelium, but when it was grown in trehalose medium, trehalose in the mycelium was not common. In our experimental conditions, we could not find a correlation between the accumulation of trehalose and stress tolerance.

### Impact of Trehalose on the Lipid, Fatty Acid and Phospholipid Profiles of *C. echinulata* FR3

On the 8th day, the total lipid content reached 25.11% in 2% trehalose medium, which was lower than those in 5% trehalose (32.29%) and 5% glucose (34.05%) media (Supplementary Figure S3). The time course of fatty acid compositions is presented in **Table 1**. The GLA content in the 2% trehalose medium was 18.63% on the 7th day and 21.92% on the 8th day, higher than those in the 5% glucose and 5% trehalose media. The GLA content in the 2% trehalose medium increased 23.2%, 65.8% higher than that in 5% glucose, on the 7th and the 8th day. The GLA content in the 5% trehalose medium increased 6.5% on the 7th day and 31.4% on the 8th day. These results demonstrated that trehalose promoted GLA synthesis in *C. echinulata* FR3.

**Table 1:** The total fatty acid profiles of C. echinulata FR3 using 5% glucose, 2% trehalose, and 5% trehalose as carbon sources.

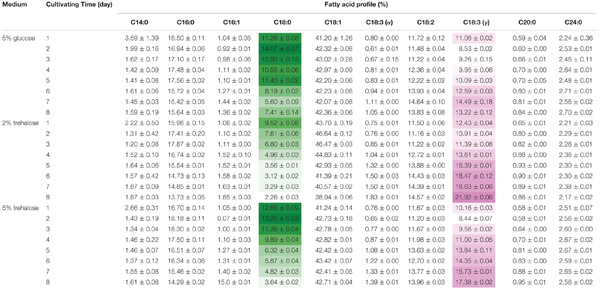

*Green/Red: the deep color means “high content,” and the light color means “low content.”*

**FIGURE 3:**
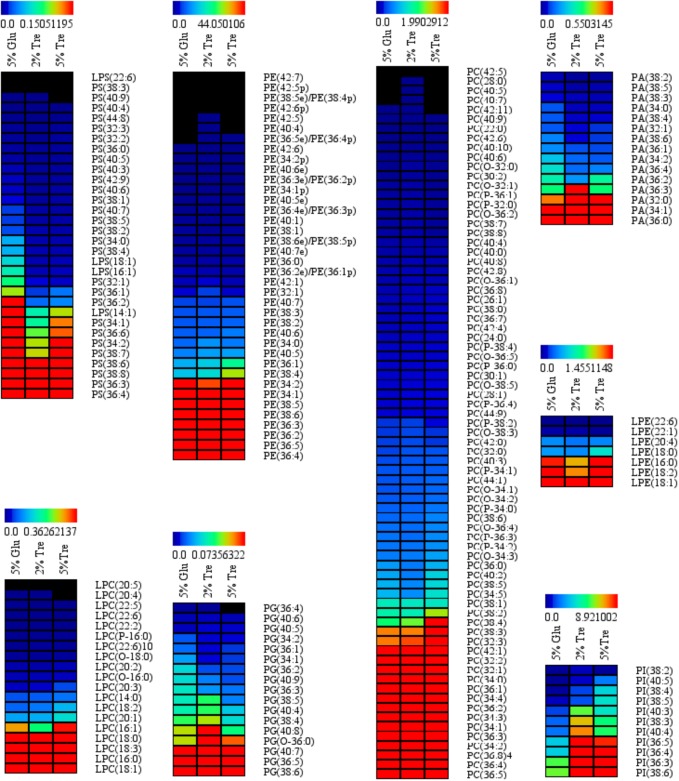
Phospholipid (PL) abundance in *C. echinulata* FR3 using 5% glucose, 2% trehalose, and 5% trehalose as carbon sources.

Phospholipids provide the permeable barrier that protects the cell and intracellular organelles, form membrane surfaces for biochemical reactions catalyzed by membrane-bound proteins, perform signal transduction in response to internal or external stimuli, and act as precursors for the synthesis of other bioactive molecules (Vance and Vance, 2004). The influence of trehalose on the PLs of *C. echinulata* FR3 was evaluated by MS-based lipidomics. On the 7th day, total PLs was 6.93% in the 2% trehalose medium and 7.28% in the 5% trehalose medium, both of which were slightly higher than that in the 5% glucose medium (6.15%). In total, 214 species of PLs were detected, including 38 phosphatidylethanolamines (PEs), 7 lysophosphatidylethanolamines (LPEs), 75 phosphatidylcholines (PCs), 19 lysophosphatidylcholines (LPCs), 25 phosphatidylserines (PSs), 4 lysophosphatidylserines (LPSs), 15 phosphatidic acids (PAs), and 11 phosphatidylinositols (PIs). The abundance, content and fatty acid composition of the PLs are presented in **Figure 3** and **Tables 2**, **3**. PE and PC were the two major classes of PLs in *C. echinulata* FR3. PCs are mainly localized to the outer membrane and function in membrane-mediated cell signaling, while PEs are mainly localized to the inner membrane and play a role in membrane architecture. The phospholipid profiles in the trehalose and glucose treatment groups were highly similar except for the PIs, and the concentration of trehalose did not affect the phospholipid profile. The PI composition of the PLs reached 5.99% in the 2% trehalose medium and 4.34% in the 5% trehalose medium, which was twofold that in the 5% glucose medium. Among them, the contents of PI (36:4), PI (36:3), PI (36:5), PI (38:3), PI (38:6), and PI (40:5) increased, and polyunsaturated acyl chains were abundant. PI is a soluble inositol phosphate and a metabolic precursor of phosphoinositides; it is synthesized in the endoplasmic reticulum (ER) and serves as a means to supply lipids to other membranes, transfer proteins and regulate lipid biology (Balla, 2013; Grabon et al., 2015). Trehalose might trigger the PI-related signal transduction pathway. To understand the promotion of GLA content, the samples of *C. echinulata* FR3 (2% trehalose and 5% glucose) on the 7th day were examined to reveal the transcriptional mechanism underlying the effects of trehalose on lipid and GLA synthesis (**Table 1**).

**Table 2:** Phospholipids and their proportions (%) in C. echinulata FR3 using 5% glucose, 2% trehalose, and 5% trehalose as carbon sources.

Lipid class	Numbers	Composition (%, w/w)			Folds		
			
		5%Glu	2%Tre	5%Tre	5%Glu vs. 2%Tre	5%Glu vs. 5%Tre	2%Tre vs. 5%Tre
LPE	7	0.40	0.35	0.38	1.10	0.87	0.79
PE	38	66.23	66.15	63.36	0.95	0.85	0.89
PG	17	0.10	0.09	0.04	1.06	2.07	1.95
LPS	4	0.03	0.00	0.00	40.62	22.06	0.54
PS	25	0.59	0.12	0.10	4.69	3.78	0.81
PA	15	0.30	0.24	0.19	1.17	1.28	1.10
PI	11	1.74	5.99	4.34	0.28	0.33	1.18
LPC	19	0.89	0.65	0.99	1.31	0.73	0.56
PC	75	29.71	26.40	30.58	1.07	0.79	0.74
PC/PE		0.45	0.40	0.48			
(LPC + PC)/(LPE + PE)		0.46	0.41	0.50			
Total phospholipid (g/100 g biomass)		6.15 ± 0.01	6.93 ± 0.01	7.28 ± 0.13			
Total phospholipid (g/100 g total lipid)		18.07 ± 0.80	27.59 ± 0.01	22.55 ± 6.54			


*LPE, lyso-phosphatidylethanolamine; PE, phosphatidylethanolamine; PG, phosphatidylglycerol; LPS, lyso-phosphatidylserine; PS, phosphatidylserine; PA, phosphatidic acid; PI, phosphatidylinositol; LPC, lyso-phosphatidylcholine; PC, phosphatidylcholine; 5%Glu, 5% glucose; 2%Tre, 2% trehalose; 5%Tre, 5% trehalose.*

**Table 3:** The composition of phospholipids and total lipids in 7-day C. echinulata FR3 using 5% glucose, 2% trehalose, and 5% trehalose as carbon sources.

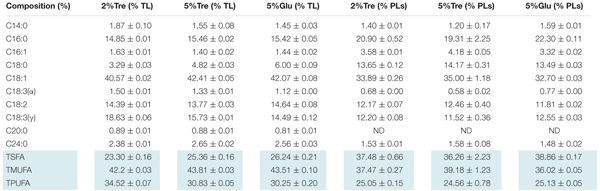

*TL, total lipids; PLs, phospholipids; ND, not detection.*

### RNA-Seq, *de Novo* Assembly, and Transcriptome Annotation

For each sample, approximately 37 million clean reads containing a total of 5G nucleotides were obtained. The GC content was 35%. FastQC analysis showed that 91% of the total sequences were of quality above Q30. The data were uploaded to the Sequence Read Archive database in NCBI (accession number SRP144437).

A total of 58,454 transcripts were assembled *de novo* with an N50 length of 1316 bp, and 39,363 unigenes were obtained with an N50 length of 1,040 bp using Trinity (Grabherr et al., 2011) (Supplementary Table S2). The annotation showed that only 48.63% of the unigenes in the transcriptome of *C. echinulata* FR3 were matched in a BLASTx search (*E*-value ≤ 1.0e-05), suggesting low similarity with other species. The remaining 51.39% unmatched unigenes might reflect genomic diversity (**Table 4**). The length distributions of *C. echinulata* transcripts (Supplementary Figure S4A) and unigenes (Supplementary Figure S4B) indicated that most unigenes (37,463, 95.18%) had fewer than 2,000 nucleotides. Based on their *E*-values, 31.3% of the matched sequences had strong homology with the database sequences (1.0e-60), and 68.7% sequences had lesser homology with the database sequences (1.0e-5 to 1.0e-60). Based on the similarity distribution, 47.6% of the matched unigenes had strong similarity to the database sequences (>80%), while 52.4% unigenes had lesser similarity to the database sequences (18% < x < 80%) (Supplementary Figure S4C). The distribution of the species with the highest sequence similarity showed that the top hits for 12.9% of unigenes were from *Absidia idahoensis*, followed by *Rhizopus microsporus* (9.8%) (Supplementary Figure S4D).

**Table 4:** Annotation statistics of C. echinulata FR3 unigenes.

	Number of unigenes	Percentage (%)
Annotated in NR	19144	48.63
Annotated in NT	10551	26.8
Annotated in KO	7596	19.29
Annotated in SwissProt	14639	37.18
Annotated in PFAM	14863	37.75
Annotated in GO	15226	38.68
Annotated in KOG	9966	25.31
Annotated in all databases	2463	6.25
Annotated in at least one database	26591	67.55
Total unigenes	39363	100


### Functional Classification of C. *echinulata* Unigenes

The functions of the *C. echinulata* unigenes were classified by GO analysis (Quevillon et al., 2005). A total of 15226 unigenes were successfully annotated with GO terms and were classified into three major GO categories: Biological Process (BP), Cell Component (CC), and Molecular Function (MF). The unigenes were further assigned to 54 subcategories based on GO level 2. The dominant subcategories for the classified genes were “cell” (18.9%) and “cell part” (18.9%) for the CC category; “cellular process” (22.3%), “metabolic process” (21.15%) and “single-organism process” (17.64%) for the BP category; and “binding”(43.8%), “catalytic activity” (38.2%), and “transporter activity” (7.2%) for the MF category (Supplementary Figure S5).

KEGG analysis was carried out to identify the biochemical pathways active in *C. echinulata* FR3. The KEGG-annotated unigenes (7,596) were attributed to 262 pathways. A total of 889 unigenes (11.7%) were involved in “translation,” and 851 unigenes (11.2%) were involved in “carbohydrate metabolism” (Supplementary Figure S6).

**FIGURE 4:**
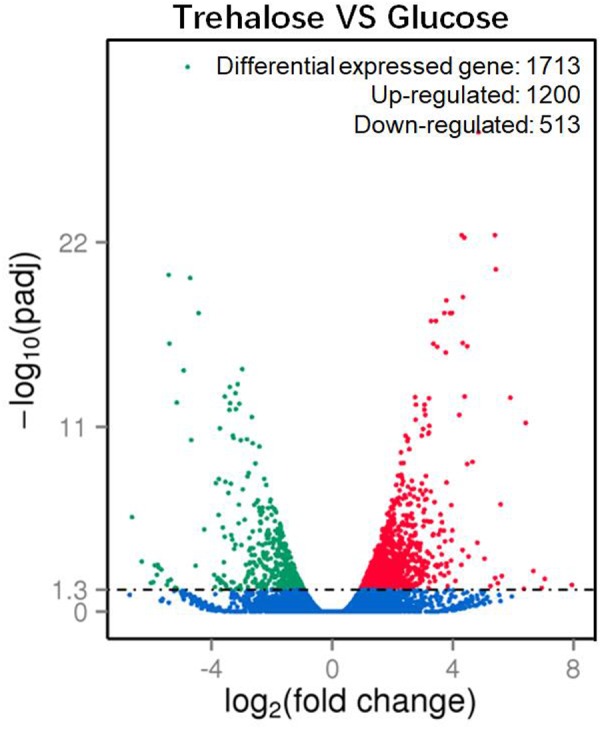
Volcano plot of significantly up-/downregulated genes in *C. echinulata* FR3 using 2% trehalose and 5% glucose as carbon sources.

A total of 1713 unigenes with significantly different expression levels were identified by sequence alignment. Of these, 1200 unigenes were upregulated, and 513 unigenes were downregulated in 2% trehalose medium compared with their expression in 5% glucose medium (**Figure 4**). These genes were involved in many lipid-related pathways. The upregulated pathways were the PPAR signaling pathway, peroxisome, cAMP signaling pathway, and endocytosis, while the downregulated pathways were the biosynthesis of amino acid and starch and sucrose metabolism (Supplementary Figure S7). The expression of some genes in 2% trehalose medium were validated by qRT-PCR (Supplementary Figure S8). These results showed that many lipid-related metabolic pathways were significantly upregulated in trehalose medium.

### Pathways Related to Sugar Metabolism

**FIGURE 5:**
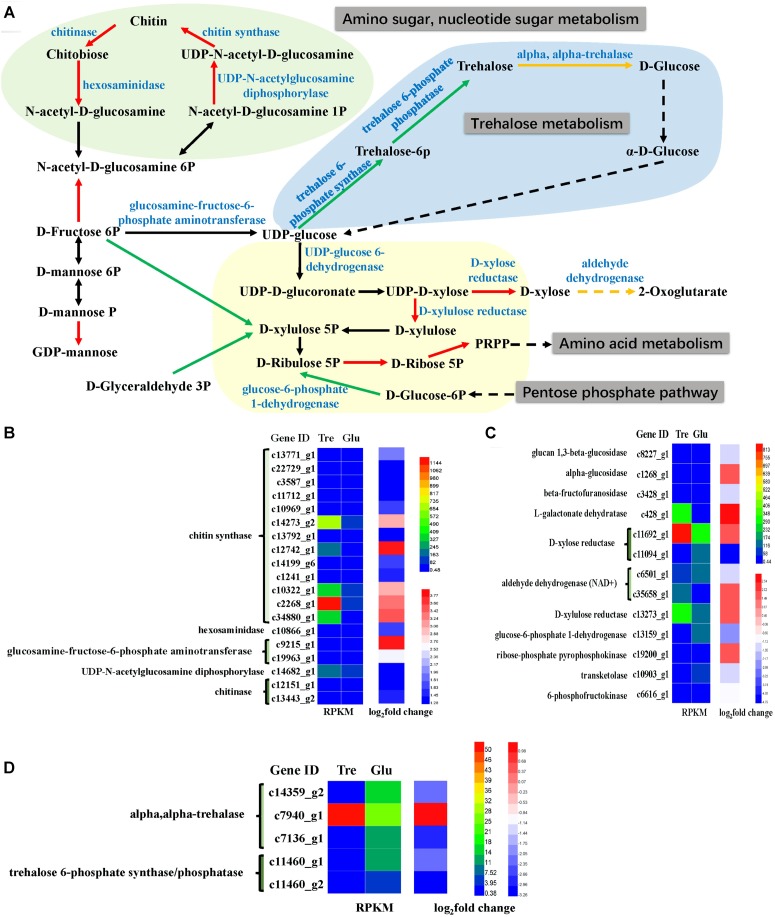
Analysis of sugar metabolism. **(A)** A constructed map of sugar metabolism responses to trehalose; **(B)** chitin-related genes (light green section); **(C)** xylose-related genes (light yellow section); **(D)** trehalose-related genes (light blue section).

Many carbohydrate metabolic pathways, such as the starch and sucrose metabolic pathways and the glycolysis and pentose phosphate pathways, were downregulated (**Figure 5A** and Supplementary Table S3). The genes encoding glucose-6-phosphate dehydrogenase (G6PD) in the pentose phosphate pathway, which is also part of the glutathione metabolic pathway, was downregulated (**Figure 5C**), while isocitrate dehydrogenase was upregulated. These two enzymes are considered NADPH suppliers. Usually, G6PD is considered the main source of NADPH for lipid synthesis, and the lipid content of the 2% trehalose medium on the 7th day was lower than that in the 5% glucose medium because of G6PD downregulation. Moreover, D-xylose reductase and D-xylulose reductase, which are related to xylose metabolism, were upregulated. It has been reported that the GLA content of mycelia grown in xylose medium was somewhat higher than that in mycelia grown with other carbon sources (Fakas et al., 2009). Chitin synthase and UDP-*N*-acetylglucosamine diphosphorylase, which are involved in amino sugar and nucleotide sugar metabolism, were upregulated (**Figure 5B**), suggesting that they were active when trehalose was used for fungal growth. In fact, Levin (2005) have found that trehalose is closely related to the cell wall integrity signaling pathway. These results indicated that trehalose induced chitin- and xylose-related metabolism. The trehalose-metabolism-related enzymes trehalose 6-phosphate phosphatase, trehalose 6-phosphate synthase, and α,α-trehalase were downregulated (**Figure 5D**). This downregulation of trehalose metabolism might be the reason for the gradual decrease in trehalose.

### Pathways Related to Lipid Metabolism

**FIGURE 6:**
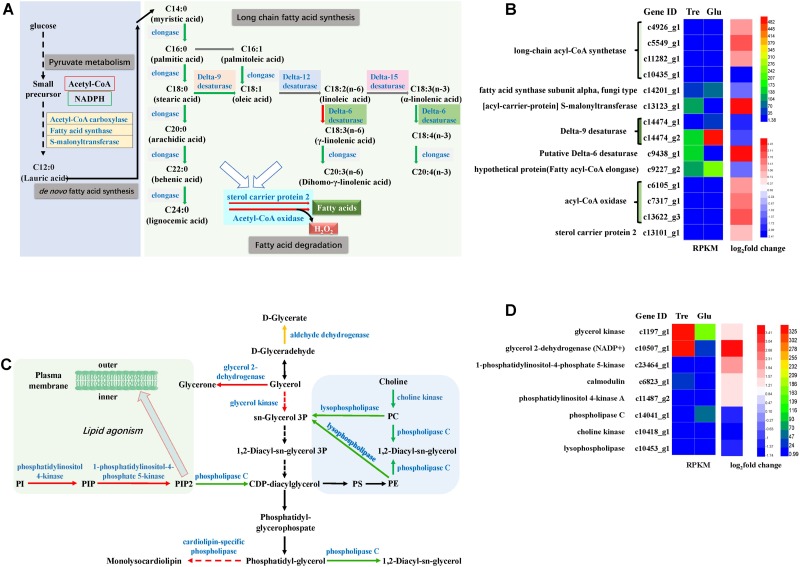
Analysis of fatty acid metabolism and phospholipid metabolism. **(A)** A constructed map of fatty acid metabolism responses to trehalose; **(B)** fatty acid-related genes; **(C)** a constructed map of phospholipid metabolism responses to trehalose; **(D)** phospholipid-related genes.

The pathway of unsaturated fatty acid synthesis was constructed in **Figure 6A** based on our transcriptomic analysis. Fungal fatty acid synthases involved in *de novo* fatty acid biosynthesis were downregulated (**Figure 6B** and Supplementary Table S3), which might be attributed to an insufficiency of the carbon source when the fungus is grown in 2% trehalose. This result agreed with the results of the lipidomic analysis (Supplementary Figure S2). Delta-9 desaturase and delta-12 desaturase were downregulated, while acyl-CoA oxidases and delta-6 desaturase were significantly upregulated (**Figure 6B**). These enzymes are critical to the biosynthesis of unsaturated fatty acids. Moreover, GLA is synthesized from LA by delta-6 desaturase, which is the rate-limiting enzyme in the essential fatty acid cascade (Buist, 2004). GLA content increased with the increasing expression of delta-6 desaturase in trehalose medium. Even though the other fatty acid desaturases were downregulated, their initial activities were higher than that of delta-6 desaturase, and therefore, they provided enough precursor molecules for GLA synthesis. Moreover, long-chain acyl-CoA synthetase, an enzyme involved in the biosynthesis of fatty acids, was remarkably increased, suggesting that this enzyme might play an important role in supplying acetyl-CoA for unsaturated fatty acid synthesis in trehalose medium.

**FIGURE 7:**
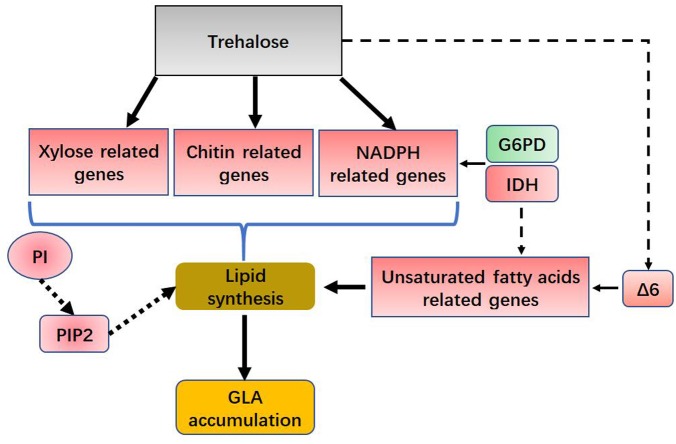
Hypothetical role for trehalose in lipid and GLA accumulation. G6PD, glucose-6-phosphate dehydrogenase; IDH, isocitrate dehydrogenase; Δ6, delta-6 desaturase; PI, phosphatidylinositol; PIP2, Phosphatidylinositol 4,5-bisphosphate.

The pathway of phospholipid synthesis was also constructed in **Figure 6C** based on our transcriptomic analysis. PI accumulation and composition variation were confirmed by phospholipidomic analysis (**Table 2**). 1-Phosphatidylinositol-4-phosphate 5-kinase (PI4P5K) and phosphatidylinositol kinase were significantly upregulated (**Figure 6D** and Supplementary Table S3), which would induce additional PIP_2_ (a form of PI) production. Takatori et al. (2014) used a method called quick-freezing and freeze-fracture labeling to obtain the distribution of lipids in the cellular membrane and verified that PI(4,5)P_2_ was enriched in the cytoplasmic leaflet (inner) of the gap junction membrane. PI(4,5)P2 is the most abundant PI and plays roles in membrane-cytoskeleton interaction, membrane trafficking, and regulation in ion channels (Takatori et al., 2014). Takatori also found that PIs enriched with polyunsaturated acyl chains might not be compatible with lipids enriched with saturated acyl chains (Takatori et al., 2014) (**Figure 6C**). Thus, we proposed a hypothetical pathway for GLA accumulation in *C. echinulata* FR3 based on our results (**Figure 7**).

## Discussion

Many reports exist about the functions of trehalose in fungal biology (Rubio-Texeira et al., 2016; Argüelles et al., 2017). First, trehalose is a reserve carbohydrate and energy source used in energy storage for fungal spores and during germination (Thevelein, 1984). In the process of yeast ascus germination, up to 80% of the trehalose is transformed into glucose during the initial stages of germination. However, the remaining 20% of the trehalose is retained as a pool in special cellular compartments (Barton et al., 1982). Second, trehalose is a stabilizer or chemical chaperone of membranes and proteins (Yancey, 2005), exerting its effect by preventing protein denaturation, genetic damage, and membrane disruption during stress (Eleutherio et al., 2015). Trehalose is also a major compatible solute in cells (Zou et al., 2017). Third, trehalose is a cell protectant under various types of stress (Elbein, 1974); it can lower the oxidative stress level, activate autophagy, and upregulate antioxidant gene expression (Mizunoe et al., 2018). Fourth, trehalose is a regulator of glycolysis and the intracellular concentrations of glucose and ATP. When excess glycolysis occurs, trehalose biosynthesis serves as a metabolic buffer (Tereshina, 2005; Argüelles et al., 2017). Finally, trehalose is a virulence factor, and accumulating evidence supports this point (Schwarz and Van Dijck, 2017; Zeidler et al., 2017; Collins et al., 2018). Given the above, trehalose might be considered a multifunctional sugar.

In our study, trehalose as a sole carbon source can provide energy for fungal growth and lipid synthesis. We found that trehalose promoted GLA production compared with glucose. In addition to its role as a carbon and energy source, other functions of trehalose in lipid and GLA synthesis include a role as a fatty acid protector, which has been validated in model organisms (de Jesus Pereira et al., 2003; Herdeiro et al., 2006) and occurs as follows.

**FIGURE 8:**
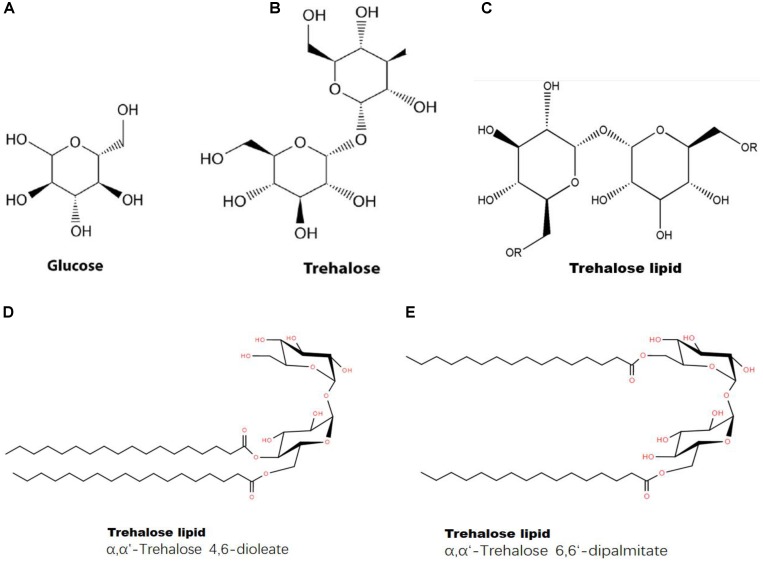
Structures of glucose, trehalose and a trehalose-lipid complex. **(A)** Chemical structure of glucose; **(B)** chemical structure of trehalose; **(C)** chemical structure of trehalose-lipid (“R” represents lipid); **(D)** Trehalose-lipid (α, α′-Trehalose 4,6-dioleate); **(E)** Trehalose-lipid (α, α′-Trehalose 6,6-dipalmitate).

First, trehalose can combine with a lipid to form a trehalose-lipid complex (**Figure 8**). The molecular structure of trehalose is more flexible than those of other disaccharides. The presence of a glycoside bond makes it possible to bind to irregularly distributed polar groups of macromolecules (Yatsyshyn et al., 2017). A trehalose-lipid complex is a glycolipid biosurfactant, esterified by two or three hydroxyls and branched fatty acids at both the 6- and 6’-OH positions from trehalose (Franzetti et al., 2010). This kind of complex has received increasing attention, because of its unusually strong immunomodulation and anticancer potentials and its utility for bioremediation (Franzetti et al., 2010; Paulino et al., 2016). So far, trehalose-lipids are known to be produced by several gram-positive bacteria, particularly an oleaginous bacterium, *Rhodococcus* sp. (Lang and Philp, 1998; Paulino et al., 2016). A trehalose-lipid complex could serve as a component of the cell wall in microorganisms (Yatsyshyn et al., 2017). Trehalose can prevent autooxidation of unsaturated acids by stoichiometrically interacting with one *cis*-olefin double bond of an unsaturated fatty acid during simultaneous dehydration *in vitro*, forming a stable complex and leading to a significant reduction in oxidation levels (Oku et al., 2003); this process prevents the degradation of unsaturated fatty acids (da Costa Morato Nery et al., 2008). Additionally, trehalose can reduce oxidative damage in cells by scavenging free radicals, which protects lipids against free radical oxidation (de Jesus Pereira et al., 2003; Mizunoe et al., 2018). Therefore, we concluded that trehalose could directly interact with lipids and stabilize the structures of fatty acids.

Second, trehalose plays a role in protecting cells from oxidative injuries, freezing, dehydration, heat stress and nutrient starvation, especially by stabilizing membranes (Crowe et al., 1987; Benaroudj et al., 2001). Trehalose preserves lipid bilayers during dehydration and rehydration by replacing water to form hydrogen bonds between its own OH groups and lipid headgroups, which maintains membrane integrity (Herdeiro et al., 2006). How does trehalose interact with the lipid bilayer? Mathematical molecular modeling and physicochemical studies using X-ray diffraction and solid-phase NMR show that trehalose forms hydrogen bonds with the polar heads of phospholipids (Toke et al., 2004). Additionally, the sugar intercalates into the membrane, performing the function of a spacer and increasing the distance between phospholipid molecules (Lee et al., 1989; Villarreal et al., 2004). Researchers have investigated the interactions between lipid membranes and trehalose using molecular dynamics. They find that trehalose concentration affects the compressibility modulus of the membrane. The interactions between the lipid bilayer and biomolecules play a role in membrane curvature generation and sensing. Trehalose prefers regions with high curvature, where it provides a favorable situation for lipid-sugar interactions.

Our results showed for the first time that trehalose induced GLA accumulation *in vivo*, which might be attributed to increases in GLA synthesis and GLA stability. First, trehalose upregulated delta-6 desaturase, increasing GLA synthesis; second, the GLA content of the PLs changed little, which meant that the increased GLA was in glycolipids or lipid droplets. Trehalose can combine with lipids and form different glycerol lipids with different degrees of unsaturation; it prefers to combine with double-unsaturated fatty acids over monounsaturated fatty acids (Kügler et al., 2014). Whether trehalose tends to combine with polyenes, increasing GLA stability, is unclear.

We also wondered whether trehalose had any structural interaction with delta-6 desaturase. To the best of our knowledge, few reports exist about the interaction of trehalose with proteins. Some researchers claim that trehalose-protein is important for cell wall biosynthesis (Paul et al., 2008), not only for its role in carbon metabolism regulation but also for direct physical interactions with cell wall biosynthetic enzymes, and linking trehalose with cell wall biosynthesis may uncover potential novel antifungal targets (Thammahong et al., 2017). Based on our transcriptomic analysis, we found that the expression levels of chitin-related enzymes and delta-6 desaturase were upregulated when trehalose was used as a carbon source. For a long time, trehalose was considered a storage carbohydrate in fungi (Rubio-Texeira et al., 2016). Trehalose has since been found to be a multifunctional molecule, and many genes are involved in trehalose metabolism. Some roles have been verified (Zeidler et al., 2017), but many issues remain unclear (Argüelles, 2017; Ash, 2017), particularly the uncertain potential interaction between trehalose and polyenes or PUFAs in the spatial structure of the membrane (Oku et al., 2003). In addition, the interactions between trehalose and delta-6 desaturase, whether direct or via a signaling cascade, must be further explored. Further studies of the mechanism by which trehalose regulates lipid and PUFA synthesis, including the interactions between trehalose and polyenes and trehalose and delta-6 desaturase are under investigation.

In summary, we showed that trehalose contributed to GLA accumulation in *C. echinulata* FR3, and in this paper, we studied that how the fungus achieves that effect. Based on lipidomic and transcriptomic analyses, trehalose upregulated delta-6 desaturase expression, increasing GLA synthesis. In addition, PI accumulated and preferred polyunsaturated acyl chains, which is beneficial for the delivery of GLA to the cytoplasm. These results will provide the basis for exploring further functions of trehalose in PUFA production.

## Author Contributions

SL and FM designed the research. SL and QY performed the experiments, analyzed and interpreted the data. SL, FM, and XZ wrote the manuscript. JY and SZ conducted a portion of the laboratory work. XZ and FM provided financial support. All authors read and approved the final manuscript.

## Conflict of Interest Statement

The authors declare that the research was conducted in the absence of any commercial or financial relationships that could be construed as a potential conflict of interest.
