# Supplementary material for: Trehalose Contributes to Gamma-Linolenic Acid Accumulation in Cunninghamella echinulata Based on de Novo Transcriptomic and Lipidomic Analyses
Source: Front Microbiol. 2018 Jun 15;9:1296. doi: 10.3389/fmicb.2018.01296 (PMC6013572; doi:10.3389/fmicb.2018.01296)
Supplement: Supplementary file 1 [file Presentation_1.PDF]

Supplementary Material

Trehalose contributes to gamma-linolenic acid accumulation identified by de novo transcriptome and lipidome analyses of *Cunninghamella echinulata* FR3

Shue Li, Qiang Yue, Shuai Zhou, Jing Yan, Xiaoyu Zhang and Fuying Ma*

Correspondence: Fuying ma

mafuying@hust.edu.cn

1. Supplementary Figures and Tables

1.1 Supplementary Figures

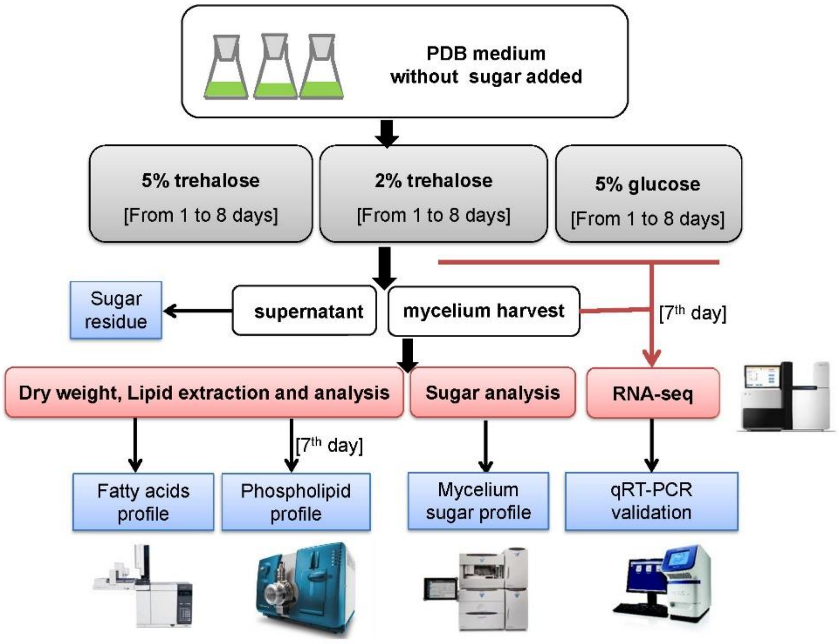

Figure S1 The experimental flow chart of our study

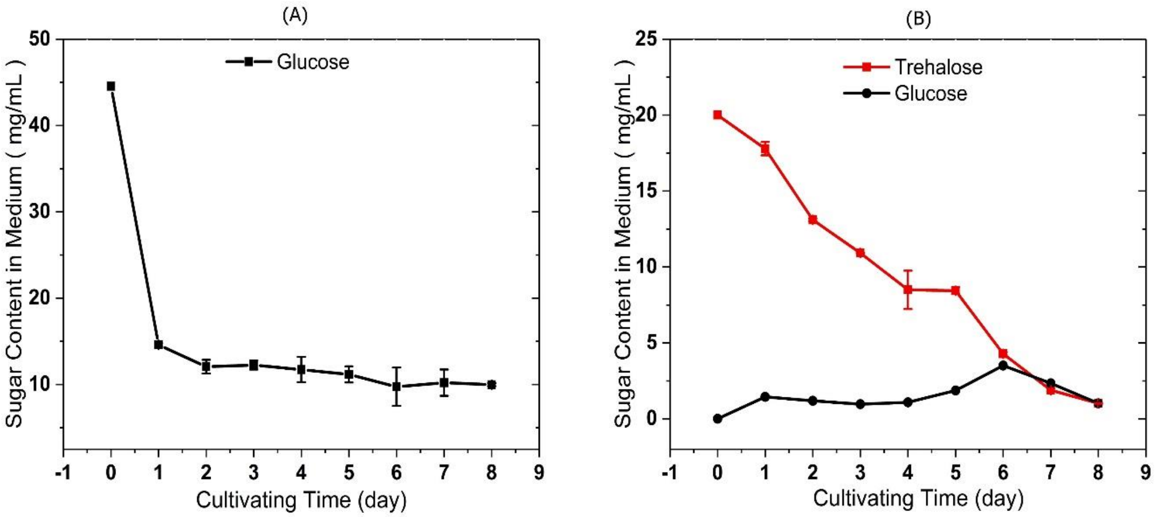

Figure S2. Time courses of changes in concentration of glucose and trehalose in culture supernatant of *C. echinulata* FR3 using 5% glucose (A) and 2% trehalose (B) as carbon source

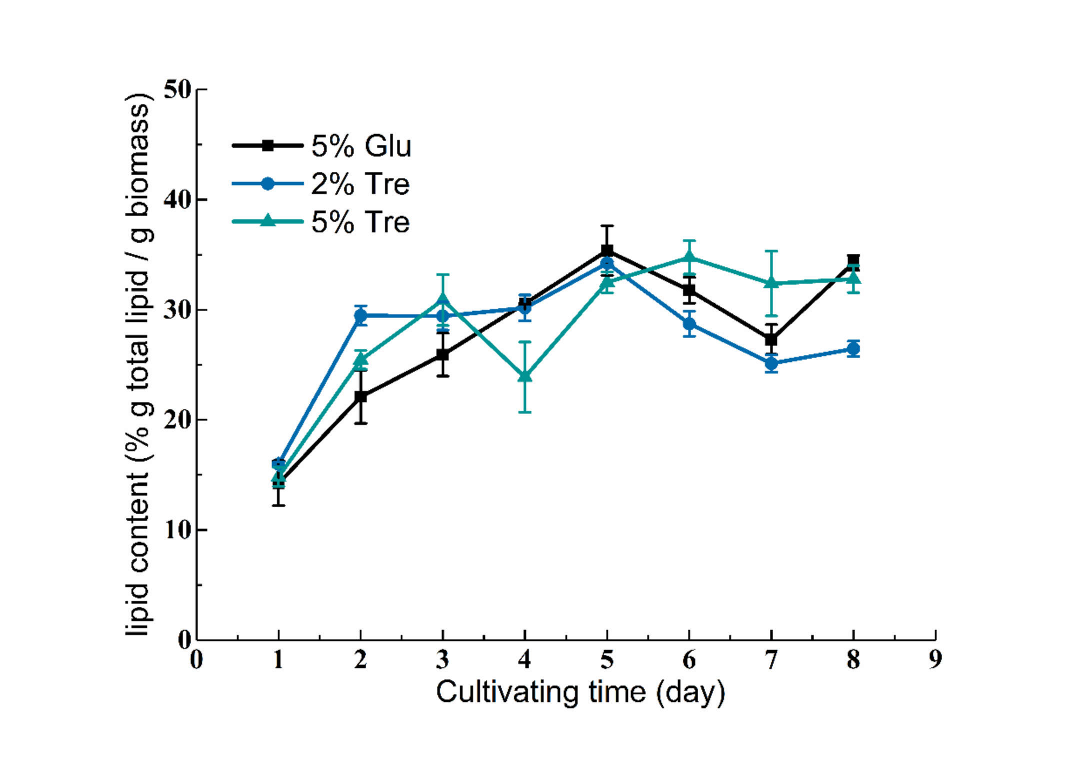

Figure S3. Time course of lipid content of *C. echinulata* FR3 using 5% glucose, 2% and 5% trehalose as carbon source, respectively.

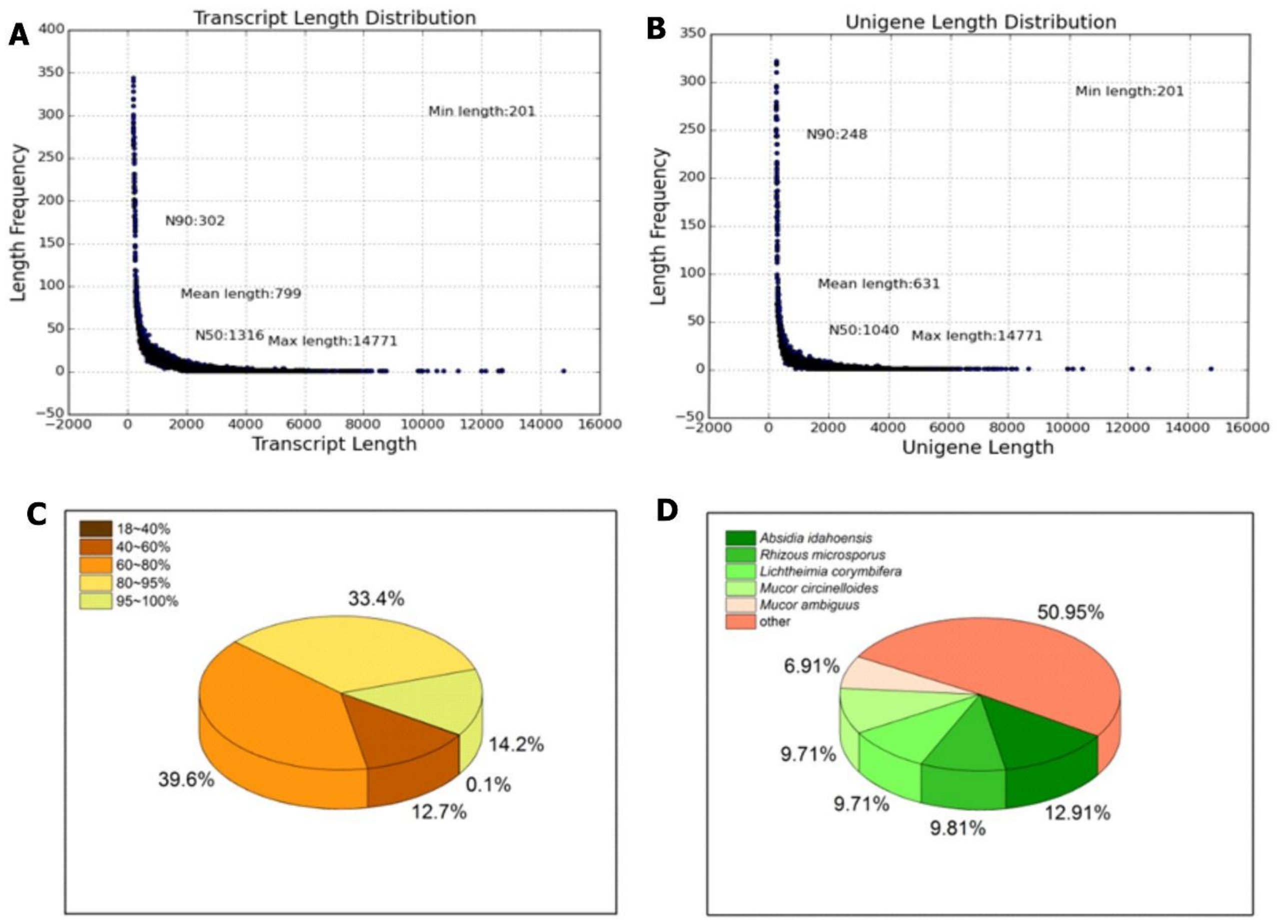

Figure S4. Transcript (**A**) and unigenes (**B**) distribution of *C.echinulata* FR3. E-value distribution (**C**) and species distribution (**D**) based on the top BLASTX hits of *C.echinulata* FR3.

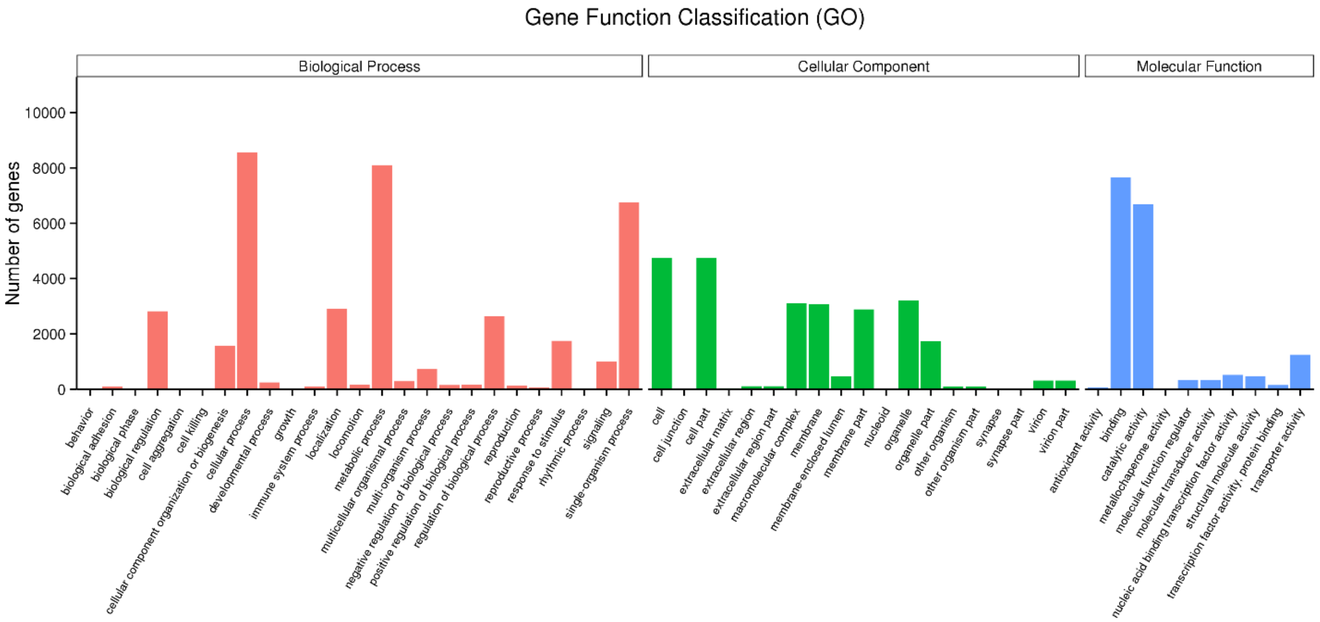

Figure S5. Gene function classification (GO) of *C. echinulata* FR3

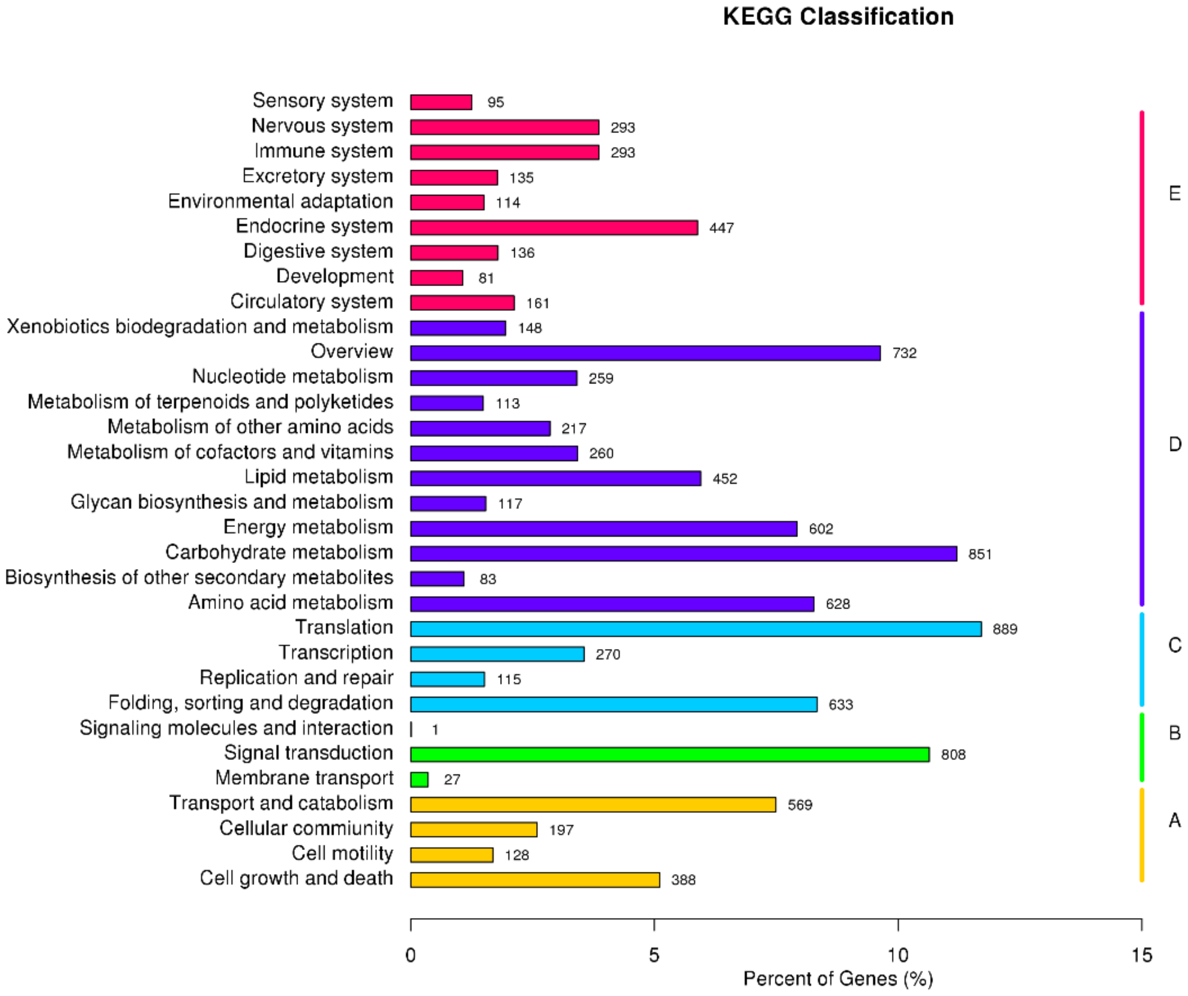

Figure S6. KEGG classification of *C. echinulata* FR3

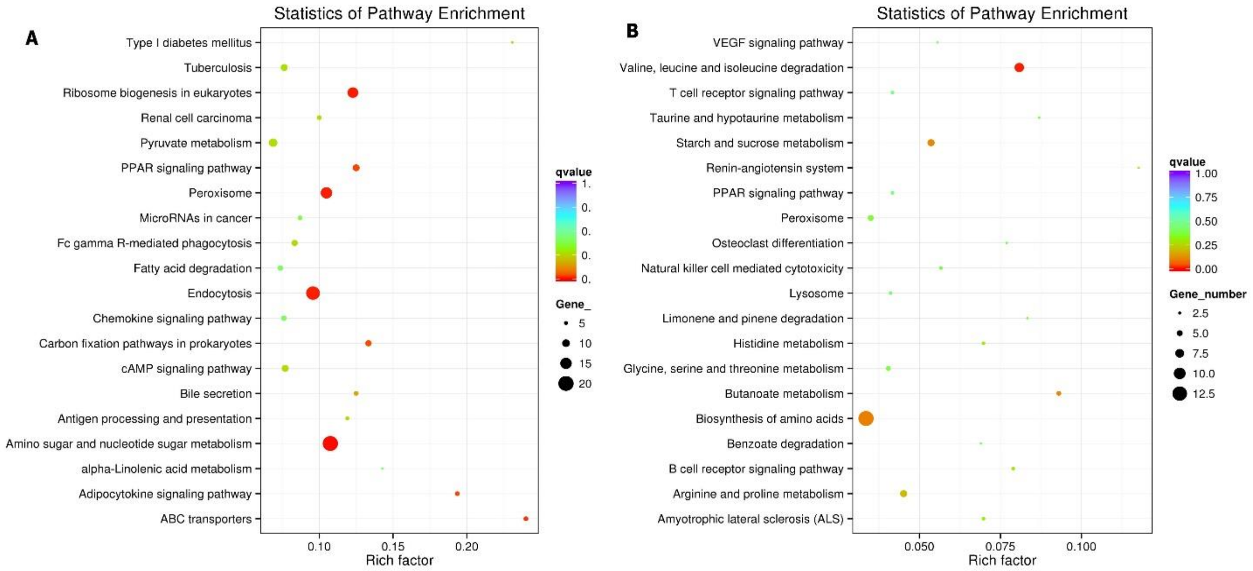

Figure S7. Statistics of pathway enrichment. A: up-regulated; B: down-regulated.

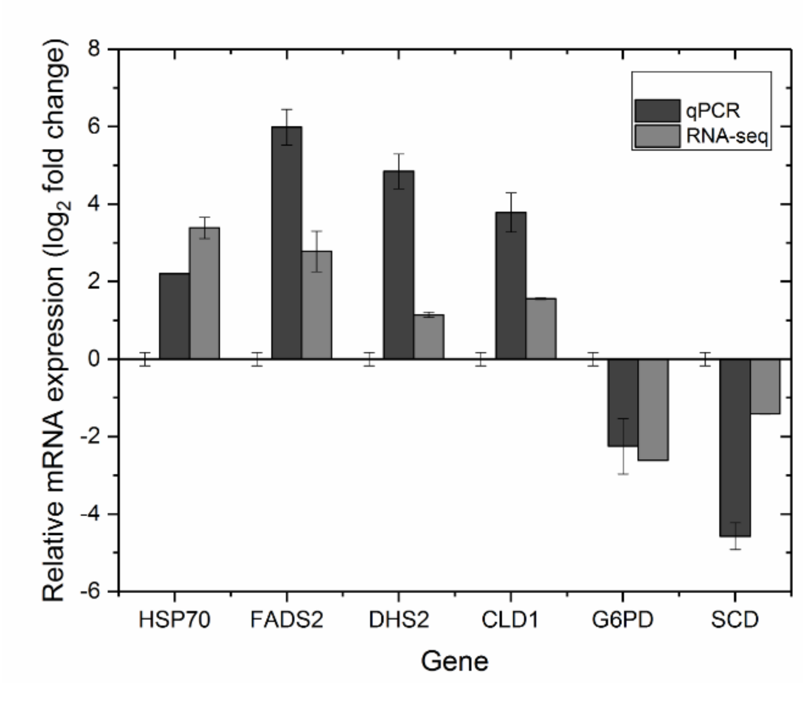

Figure S8. qPCR validation of differentially expressed genes related to lipid synthesis of *C. echinulata* FR3 using 2% trehalose as carbon source. The qRT-PCR data represents the mean ±SD of the three replicates.

1.2 Supplementary Tables

Table S1. Information on genes, primers and primer sequences (5' -3') used in the qRT-PCR analysis

Transcript ID	Gene	Forward	Reverse	Abbreviation
c14455_g1	18S (reference gene)	TCGGAAGGGGTGCACTTATT	ACCCTAATTCCCCGTTACCC	18S
c14604_g5	Putative Heat shock protein 70	GGTGTTAACAAAGATGCGCGAAA	CGGCGGCAGTAGGTTCATTAATA	HSP70
c9228_g1	Putative Delta-6 desaturase	ATTGCTGATCATCCTGGTGGTG	AAGGTGGAGCCTTCAAAATAACC	FADS2
c14385_g2	3-deoxy-7-phosphoheptulonate synthase	CGATTCCTCATCCACCACTT	TTGTACGATCCCCAATCCAT	DHS2
c8291_g1	Putative Glucose-6-phosphate 1-dehydrogenase	TGACCAAGCTGATTCTTGGA	CGATTCACACCTGTTGTGCT	G6PD
c14395_g8	cardiolipin-specific phospholipase	AGCTCAAGCAGCTCCAAGAA	CCCGCTAAAGCATATTCACC	CLD1
c14474_g2	delta-9 fatty acid desaturase	GGTGGTCAAGAGGTCATCGT	AAGGATCGGCTTTCAAATCA	SCD

Table S2. Statistics of transcriptome assembly and predicted unigenes in *C. echinulata* FR3

	Min Length	Mean Length	Median Length	Max Length	N50	N90	Total Nucleotides
Transcripts	201	799	461	14771	1316	302	46681599
Unigenes	201	631	338	14771	1040	248	24850180

Table S3. Pathways encompassed by the differentially expressed genes in the transcriptome between trehalose and glucose. “(n)” means the number of unigene

pathway	Differentially expressed gene	
	**Up-regulated**	**Down-regulated**
Carbohydrate metabolism related pathways		
Starch and sucrose metabolism	EC3.2.1.28- alpha,alpha-trehalase (1)<br>EC3.2.1.20- maltase-glucoamylase (1)	EC3.2.1.28- alpha,alpha-trehalase (2)<br>EC3.1.3.12- trehalose 6-phosphate phosphatase (2)<br>EC2.4.1.15- trehalose 6-phosphate synthase (2)<br>EC3.2.1.26- beta-fructofuranosidase (1)<br>EC3.2.1.58- glucan 1,3-beta-glucosidase (1)
Galactose metabolism	EC3.2.1.20- maltase-glucoamylase (1)	EC3.2.1.26- beta-fructofuranosidase (1)<br>EC2.7.1.11- 6-phosphofructokinase 1 (1)
Citrate cycle (TCA cycle)	EC4.1.1.49-phosphoenolpyruvate carboxykinase (ATP) (1)<br>EC4.2.1.3-aconitate hydratase (1)<br>EC1.1.1.42-isocitrate dehydrogenase (1)<br>EC1.2.4.2-2-oxoglutarate dehydrogenase E1 component (1)<br>EC4.2.1.2-fumarate hydratase, class I (3)	
Pentose and glucuronate intervconversions	EC4.2.1.146-L-galactonate dehydratase (1)<br>EC1.1.1.1.307-D-xylose reductase (1)<br>EC1.2.1.3-aldehyde dehydrogenase NAD+ (1)	EC1.1.1.1.307-D-xylose reductase (1)<br>EC1.2.1.3-aldehyde dehydrogenase NAD+ (1)
Pyruvate metabolism	EC4.1.1.49-phosphoenolpyruvate carboxykinase (ATP) (1)<br>EC2.7.1.40-pyruvate kinase (1)<br>EC1.1.2.3-L-lactate dehydrogenase (1)<br>EC6.2.1.1-acetyl-CoA synthetase (3)<br>EC1.2.1.3-aldehyde dehydrogenase NAD+ (1)<br>EC2.3.3.9-malate synthase (1)<br>EC4.2.1.2-fumarate hydratase, class II (3)	EC1.1.1.79- glyoxylate/hydroxypyruvate reductase (1)<br>EC1.2.1.3-aldehyde dehydrogenase NAD+ (1)<br>EC3.1.2.1- acetyl-CoA hydrolase (1)
Glycolysis/ Gluconeogenesis	EC2.7.1.40-pyruvate kinase (1)<br>EC4.1.1.49-phosphoenolpyruvate carboxykinase (ATP) (1)<br>EC1.2.1.3-aldehyde dehydrogenase NAD+ (1)<br>EC6.2.1.1-acetyl-CoA synthetase (3)	EC2.7.1.11- 6-phosphofructokinase (1)<br>EC1.2.1.3-aldehyde dehydrogenase NAD+ (1)<br>EC4.1.1.1- pyruvate decarboxylase (1)
Pentose phosphate pathway	EC2.7.6.1-ribose-phosphate pyrophosphokinase (1)	EC1.1.1.49- glucose-6-phosphate 1-dehydrogenase (1)<br>EC2.2.1.1- transketolase (1)<br>EC2.7.1.11- 6-phosphofructokinase (1)
Fructose and mannose metabolism	EC2.7.7.13-mannose-1-phosphate guanylyl transferase (1)	EC2.7.1.11- 6-phosphofructokinase (1)
Fatty acid metabolism-related pathways		
Fatty acid biosynthesis	EC2.3.1.39-[acyl-carrier-protein]S-malonyl transferase (1)<br>EC6.2.1.3-long-chain acyl-CoA synthetase (3)	EC2.3.1.86- fatty acid synthase subunit alpha, fungi type (1)<br>EC6.2.1.3-long-chain acyl-CoA synthetase (1)
Biosynthesis of unsaturated fatty acids	EC1.3.3.6-acyl-CoA oxidase (3)<br>EC1.14.19.3- Delta6-desaturase (1)	EC1.14.19.1- Delta9-desaturase (2)

Glycerophospholipid metabolism	EC3.1.1- cardiolipin-specific phospholipase	EC3.1.1.5- lysophospholipase I (1)<br>EC2.7.1.32- choline kinase (1)<br>EC3.1.4.3- phospholipase C (1)
Phosphatidylinositol signaling system	EC2.7.1.68- 1-phosphatidylinositol-4-phosphate 5-kinase (1)<br>EC2.7.1.67- phosphatidylinositol kinase (1)	
Glycerolipid metabolism	EC1.2.1.3-aldehyde dehydrogenase NAD+ (1)<br>EC1.1.1.156-glycerol 2-dehydrogenase (NADP+)<br>EC2.7.1.30-glycerol kinase (1)	EC1.2.1.3-aldehyde dehydrogenase NAD+ (1)
Propanoate metabolism	EC6.2.1.1-acetyl-CoA synthetase (3)	
Alpha-linolenic acid metabolism	EC1.3.3.6-acyl-CoA oxidase (3)	
Glyoxylate and dicarboxylate metabolism	EC2.3.3.9- malate synthase (1)<br>EC4.2.1.3- aconitate hydratase (1)<br>EC4.1.3.1- isocitrate lyase (1)	EC1.11.1.6-catalase (1)<br>EC1.1.1.79- glyoxylate/hydroxypyruvate reductase (1)<br>EC1.1.1.81- hydroxypyruvate reductase (1)
peroxisome	Peroxin-13 (1)<br>Protein Mpv17 (1)<br>EC1.3.3.6-acyl-CoA oxidase (3)<br>EC2.3.1.176-sterol carrier protein 2 (1)<br>EC6.2.1.3-long-chain acyl-CoA synthetase (3)<br>EC2.3.1.7-carnitine O-acetyltransferase (3)<br>EC1.11.1.6-catalase (1)<br>EC1.15.1.1-superoxide dismutase, Cu-Zn family (1)<br>EC1.1.1.42-isocitrate dehydrogenase (1)	Protein Mpv17 (1)<br>EC6.2.1.3-long-chain acyl-CoA synthetase (1)<br>EC1.4.3.3- D-amino-acid oxidase (1)<br>EC4.1.3.4- hydroxymethylglutaryl-CoA lyase (1)
PPAR signaling pathway	EC6.2.1.3-long-chain acyl-CoA synthetase (3)<br>EC2.3.1.176-sterol carrier protein 2 (1)<br>EC1.3.3.6-acyl-CoA oxidase (3)<br>Ubiquitin C (1)<br>EC2.7.1.30-glycerol kinase (1)<br>EC1.14.19.3- Delta6-desaturase (1)	EC1.14.19.1- Delta9-desaturase (2)<br>EC6.2.1.3-long-chain acyl-CoA synthetase (1)
Fatty acid degradation	EC1.3.3.6-acyl-CoA oxidase (3)<br>EC1.2.1.3-aldehyde dehydrogenase NAD+ (1)	EC1.2.1.3-aldehyde dehydrogenase NAD+ (1)<br>EC6.2.1.3-long-chain acyl-CoA synthetase (1)
Amino acid metabolism related pathways		
Cysteine and methionine metabolism	EC2.5.1.47- O-acetyl-L-serine sulfhydrylase (1)<br>EC4.4.1.1-cystathionine gamma-lyase (1)	EC1.13.11.20- cysteine dioxygenase (1)<br>EC2.7.2.4- aspartate kinase (1)
Phenylalanine, tyrosine and tryptophan biosynthesis	EC2.5.1.54-3-deoxy-7-phosphoheptulonate synthase (1)<br>EC4.2.1.20- tryptophan synthase (1)<br>EC-4.1.1.48-anthranilate synthase/indole-3-glycerol phosphate synthase (3)	EC2.5.1.54-3-deoxy-7-phosphoheptulonate synthase (1)
Glycine, serine and threonine metabolism	EC4.2.1.20- tryptophan synthase (1)<br>EC4.4.1.1-cystathionine gamma-lyase (1)	EC1.4.3.3- D-amino-acid oxidase (1)<br>EC1.1.1.79- glyoxylate/hydroxypyruvate reductase (1)<br>EC4.1.2.48- threonine aldolase (1)
Alanine, aspartate and glytamate metabolism	EC1.4.1.2-glutamate dehydrogenase (1)<br>EC2.6.1.16-glucosamine--fructose-6-phosphate aminotransferase (isomerizing) (2)<br>EC2.6.1.2- alanine transaminase (1)	EC4.3.2.1- argininosuccinate lyase (1)<br>EC4.1.1.15- glutamate decarboxylase (1)

Valine, leucine and isoleucine degradation	EC1.2.1.3-aldehyde dehydrogenase NAD+ (1)	EC2.2.1.6- acetolactate synthase (1)
Lysine degradation	EC1.2.1.3-aldehyde dehydrogenase NAD+ (1)<br>EC1.2.4.2-2-oxoglutarate dehydrogenase E1 component (1)	
Tryptophan metabolism	EC1.2.1.3-aldehyde dehydrogenase NAD+(1)<br>EC1.11.1.6-catalase (1)<br>EC1.2.4.2-2-oxoglutarate dehydrogenase E1 component (1)	EC1.2.1.3- aldehyde dehydrogenase NAD+(1)<br>EC2.6.1.27- tryptophan aminotransferase (1)<br>EC1.11.1.6-catalase (1)
Beta-alanine metabolism	EC1.5.3.14- polyamine oxidase (1)<br>EC1.5.3.16-spermine oxidase (1)	EC1.2.1.3-aldehyde dehydrogenase NAD+ (1)<br>EC4.1.1.15- glutamate decarboxylase (1)
Arginine and proline metabolism	EC1.4.1.2-glutamate dehydrogenase (1)<br>EC1.2.1.41-glutamate-5-semialdehyde dehydrogenase (1)<br>EC1.5.1.2-pyrroline-5-carboxylate reductase (1)<br>EC1.5.3.14- polyamine oxidase (1)<br>EC1.2.1.3-aldehyde dehydrogenase NAD+ (1)	EC2.3.1.1- amino-acid N-acetyltransferase (1)<br>EC2.1.3.3- ornithine carbamoyltransferase (2)<br>EC4.3.2.1- argininosuccinate lyase (1)<br>EC1.4.3.3- D-amino-acid oxidase (1)<br>EC1.2.1.3-aldehyde dehydrogenase NAD+ (1)
Valine, leucine and isoleucine biosynthesis	EC1.2.1.3-aldehyde dehydrogenase NAD+ (1)	EC1.2.4.4- 2-oxoisovalerate dehydrogenase E1 component alpha subunit (1)<br>EC2.3.1.168- 2-oxoisovalerate dehydrogenase E2 component (dihydrolipoyl transacylase) (2)<br>EC1.3.8.4- isovaleryl-CoA dehydrogenase (1)<br>EC6.4.1.4- 3-methylcrotonyl-CoA carboxylase alpha subunit (2)<br>EC4.1.3.4- hydroxymethylglutaryl-CoA lyase (1)<br>EC1.2.1.3-aldehyde dehydrogenase NAD+ (1)
Phenylalanine metabolism		EC1.1.1.157- 3-hydroxybutyryl-CoA dehydrogenase (1)
Histidine metabolism	EC3.6.1.31-phosphoribosyl-ATP pyrophosphohydrolase (1)<br>EC3.54.19-phosphoribosyl-AMP cyclohydrolase (1)<br>EC1.2.1.3-aldehyde dehydrogenase NAD+ (1)	EC4.1.3.- -cyclase (1)<br>EC2.4.2.- - glutamine amidotransferase (1)<br>EC1.2.1.3-aldehyde dehydrogenase NAD+ (1)
Other metabolic pathways		
Amino sugar and nucleotide sugar metabolism	EC3.2.1.14-chitinase (2)<br>EC3.2.1.52-beta-N-acetylhexosaminidase (1)<br>EC2.7.7.83-UDP-N-acetylglucosamine diphosphorylase (1)<br>EC2.4.1.16-chitin synthase (13)	
Purine metabolism	EC2.7.6.1- ribose-phosphate pyrophosphokinase (1)<br>EC2.7.1.40-pyruvate kinase (1)<br>EC2.7.4.8- guanylate kinase (1)	
Pantothenate and CoA biosynthesis		EC2.2.1.6- acetolactate synthase (1)
Glutathione metabolism	EC1.1.1.42-isocitrate dehydrogenase (1)	EC1.1.1.49- glucose-6-phosphate 1-dehydrogenase (1)
Oxidative phosphorylation	EC3.6.3.6- H+-transporting ATPase (1)	
